# Pulmonary artery embolism: comprehensive transcriptomic analysis in understanding the pathogenic mechanisms of the disease

**DOI:** 10.1186/s12864-023-09110-0

**Published:** 2023-01-09

**Authors:** Leszek Gromadziński, Łukasz Paukszto, Ewa Lepiarczyk, Agnieszka Skowrońska, Aleksandra Lipka, Karol G. Makowczenko, Elżbieta Łopieńska-Biernat, Jan P. Jastrzębski, Piotr Holak, Michał Smoliński, Marta Majewska

**Affiliations:** 1 Department of Cardiology and Internal Medicine, School of Medicine, Collegium Medicum, University of Warmia and Mazury in Olsztyn, Warszawska Str 30, 10-082 Olsztyn, Poland; 2 Department of Botany and Nature Protection, University of Warmia and Mazury in Olsztyn, Plac Łódzki 1, 10-727 Olsztyn, Poland; 3 Department of Human Physiology and Pathophysiology, School of Medicine, Collegium Medicum, University of Warmia and Mazury in Olsztyn, Warszawska Str 30, 10-082 Olsztyn, Poland; 4 Department of Gynecology, and Obstetrics, School of Medicine, Collegium Medicum, University of Warmia and Mazury in Olsztyn, Żołnierska Str 18, 10-561 Olsztyn, Poland; 5 Department of Animal Anatomy and Physiology, Faculty of Biology and Biotechnology, University of Warmia and Mazury in Olsztyn, Oczapowskiego 1A, 10-719 Olsztyn, Poland; 6 Department of Biochemistry, Faculty of Biology and Biotechnology, University of Warmia and Mazury in Olsztyn, Oczapowskiego Str 1A, 10-719 Olsztyn, Poland; 7 Department of Plant Physiology, Genetics and Biotechnology, Faculty of Biology and Biotechnology, University of Warmia and Mazury in Olsztyn, Oczapowskiego Str 1A, 10-719 Olsztyn-Kortowo, Poland; 8 Department of Surgery and Radiology With Clinic, Faculty of Veterinary Medicine, University of Warmia and Mazury in Olsztyn, Oczapowskiego Str 14, 10-719 Olsztyn, Poland; 9 Clinic of Cardiology and Internal Diseases, University Clinical Hospital in Olsztyn, Warszawska Str 30, 10-082 Olsztyn, Poland

**Keywords:** Pulmonary embolism, RNA-seq, Molecular pathways, Endoplasmic reticulum

## Abstract

**Background:**

Pulmonary embolism (PE) is a severe disease that usually originates from deep vein thrombosis (DVT) of the lower extremities. This study set out to investigate the changes in the transcriptome of the pulmonary artery (PA) in the course of the PE in the porcine model.

**Methods:**

The study was performed on 11 male pigs: a thrombus was formed in each right femoral vein in six animals, and then was released to induce PE, the remaining five animals served as a control group. In the experimental animals total RNA was isolated from the PA where the blood clot lodged, and in the control group, from the corresponding PA segments. High-throughput RNA sequencing was used to analyse the global changes in the transcriptome of PA with induced PE (PA-E).

**Results:**

Applied multistep bioinformatics revealed 473 differentially expressed genes (DEGs): 198 upregulated and 275 downregulated. Functional Gene Ontology annotated 347 DEGs into 27 biological processes, 324 to the 11 cellular components and 346 to the 2 molecular functions categories. In the signaling pathway analysis, KEGG ‘protein processing in endoplasmic reticulum’ was identified for the mRNAs modulated during PE. The same KEGG pathway was also exposed by 8 differentially alternative splicing genes. Within single nucleotide variants, the 61 allele-specific expression variants were localised in the vicinity of the genes that belong to the cellular components of the ‘endoplasmic reticulum’. The discovered allele-specific genes were also classified as signatures of the cardiovascular system.

**Conclusions:**

The findings of this research provide the first thorough investigation of the changes in the gene expression profile of PA affected by an embolus. Evidence from this study suggests that the disturbed homeostasis in the biosynthesis of proteins in the endoplasmic reticulum plays a major role in the pathogenesis of PE.

**Supplementary Information:**

The online version contains supplementary material available at 10.1186/s12864-023-09110-0.

## Background

Pulmonary embolism (PE) is a life-threatening disease that is one of the major causes of sudden unexpected deaths [1, 2] Most PEs originate from deep vein thrombosis (DVT), a condition occurring when a blood clot forms in the veins, typically of lower extremities (calf, femoropopliteal, and less frequently, the iliac veins) [3]. The thrombus consists mainly of fibrin, collagen, platelets and erythrocytes, and it reacts directly with the wall of the venous vessel, triggering various inflammatory processes [4]. This process is not unilateral as the endothelium exerts a feedback effect on clot formation and rearrangement [5, 6]. DVT characteristically arises in regions of reduced blood flow and then propagates as a result of hypercoagulability initiated by hypoxia and increased concentration of blood cells [1]. PE begins when the embolus disconnects from the point of origin and lodges in the pulmonary artery (PA), causing various severe consequences, including hemodynamic collapse and death [7]. When the embolic material moves into the PA, it enters a different environment. The question that arises is whether the thrombus formed in the vein will cause additional reactions of the arterial endothelium after reaching the PA, exemplary changes similar to those observed in acute coronary syndrome or ischemic stroke. When DVT and PE affect the same organism, they together constitute the disease known as venous thromboembolism (VTE) [2]. The prognosis in PE differs depending mostly on the degree of vessel obstruction, and it may lead to nonfatal VTE, fatal PE, postthrombotic syndrome, or chronic thromboembolic pulmonary hypertension [8]. However, the prognosis of PE/VTE patients is much worse than that of patients with only DVT. Treatment for both DVT and PE is similar and is targeted at preventing further increase of the clot size and new clots from forming, restoring the perfusion of the plugged vessel [9, 10]. To achieve this goal, anticoagulant therapy is widely used, initially with unfractionated or low molecular weight heparin, then, for at least 3 months, oral anticoagulants as vitamin K antagonists, or currently more preferred rivaroxaban, dabigatran, apixaban or endoxaban are used [9, 11]. In some cases of PE, fibrinolytic therapy can be applied in patients with clinical symptoms of haemodynamic instability [12]. The exact understanding of PE pathophysiology, including the knowledge of an intricate interrelationship between the PA endothelium and the lodged blood clot, helps in risk evaluation and allows for determining the most relevant and effective treatment. In the rat model, *IL‑1β*, *TF*, *XOD* and *NF‑κB* were found to be significantly differentially expressed, and thus were considered DVT biomarkers [13]. In the rabbit, it has been discovered, that genes related to the inflammation process or immune, pulmonary and cardiovascular diseases, such as *IL-8*, *TNF-α*, and *CXCL5*, may have functional importance for PE [14]. Due to multi-omics studies of the transcriptome and metabolome data of PE patients, *LIPC* and *NAT2* were found to be differentially expressed in PE [15]. Further studies investigating the molecular background of thrombus formation and its effect on the venous and arterial endothelium in course of the DVT and PE are needed. Therefore, in our previous study, we already investigated the transcriptomic profile of femoral veins in DVT, and we discovered that dysregulation of TNF, NF-κB, and apoptosis pathways affect inflammatory responses and the formation, remodeling, and resolution of thrombus in DVT pathogenesis [16]. Whereas, the present research was aimed at analyzing the changes in the gene expression associated with PE in the PA samples (PA-E). To accomplish this goal the experiment was performed in a new pig model, in which the thrombus was first formed in vivo in the femoral vein, and then was released to induce PE in the same animal, mimicking the natural way of its formation, as well as its passage through the vascular bed. As the PA-E samples were collected 3 h after the thrombus release, the changes observed in the investigated tissues most closely reflect these typical for acute PE.

## Methods

### Surgical procedures in the experimental and control animals

**Fig. 1:**
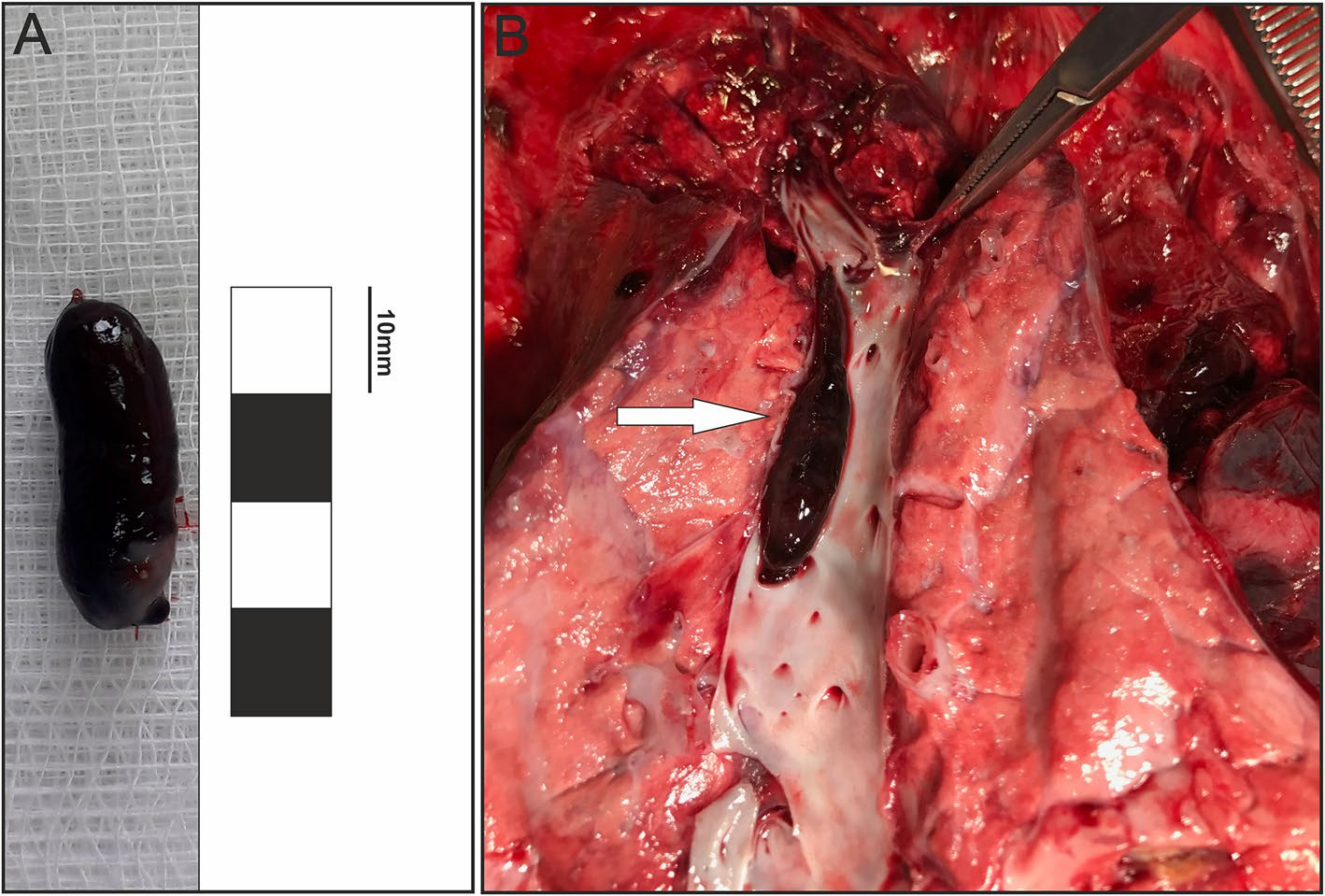
A representative images of: (**A**) an embolus collected from the segmental pulmonary artery (**B**) an embolus, indicated by a white arrow, in the segmental pulmonary artery found at autopsy

The study was performed in 11 castrated male pigs (24 weeks old, 60 kg body weight, b.w.) of the Polish Landrace breed; 6 animals served as the experimental group (further referred to as pulmonary arteries affected by embolism PA-E); the remaining 5 pigs served as controls (CTR). Several different experimental models of PE involving rats, mice, pigs and rabbits have been described [17–23]. In the present study experimental model of VTE was based on the one proposed by Robinson et al. [24] as described previously. In the experimental group first, the DVT was developed by forming the thrombus in the right femoral veins using the porcine model described previously [25]. Briefly, before performing any surgical procedures, all the DVT pigs were pretreated with atropine (Atropinum Sulfuricum, Polfa, Warsaw, Poland, 0.05 mg/kg b.w., s.c.) and azaperone (Stresnil, Janssen Pharmaceutica, Beerse, Belgium, 2.5 mg/kg b.w., i.m.). Thirty minutes later, to induce anesthesia, the main anesthetic drug propofol (Propofol-Lipuro, B. Braun Melsungen AG, Melsungen, Germany, 10 mg/kg b.w.) and the main analgesic drug ketamine (Bioketan, Vetoquinol, Poland, 10 mg/kg b.w.) were given intravenously in a slow, fractionated infusion. The depth of anesthesia was monitored by testing the corneal reflex. Once the animals were transported to an operating theater, general anesthesia during the entire procedure was maintained with inhalation of sevoflurane (Sevoflurane, Baxter, Ontario, CA USA; administered at one human MAC end-tidal concentration of 2.0%) under continuous pulse oximetry and heart-rate monitoring. Under sterile conditions, the left femoral veins were carefully exposed in each animal by dissecting the fascia and exposing and preparing the sartorius muscle. Next, the vein was closed proximally and distally at the length of about 30–40 mm with surgical ligatures. To accelerate the thrombus formation, 200 units of thrombin (BioTrombina, Biomed Lublin S.A., Lublin, Poland; the doses of the treatment agents were corresponding to those previously used in other experiments; [24]) were administered into the closed segment of the vein. Immediately afterwards, to prevent the formation of thrombi outside the closed segments, a bolus of unfractionated heparin (Heparinum WZF, Warsaw, Poland) was administered, in a dose of 100 U/kg, through a catheter inserted in the ear vein. Two hours later (an optimal time needed for thrombus formation after the closing of the femoral vein), the thrombus was released from the right femoral vein to induce PE. The thrombus release was facilitated mechanically by bending the right hind limb (thus the movements of muscles were acting as natural pistons). If this procedure was ineffective, 10 mL of saline was injected into the right femoral vein distally from the formed thrombus. Computed tomography pulmonary angiography (CTPA; Aquilion PRIME TSX-303A Toshiba, Tustin, CA, USA), a method of choice serving to assess the pulmonary vessels when PE is suspected, was performed in all experimental pigs after the release of the thrombi from the right femoral veins (Fig. 1).

Using a maximum intensity projection (MIP) technique the thrombi were visualized and measured with the Toshiba Aquilion PRIME TSX-303A as a standard software tool. During the CTPA procedure, the animals were sedated with propofol (Propofol-Lipuro, B. Braun Melsungen AG, 10 mg/kg b.w.). Three hours after the CTPA, all experimental pigs were euthanized with sodium pentobarbital (Euthasol, FATRO, Ozzano dell’Emilia BO, Italy, 140 mg/kg). The entire heart and both lungs were dissected from each animal. After the incision of the right ventricle and the right atrium, the pulmonary trunk was incised immediately above the pulmonary valve. Then, very carefully and delicately, PAs were successively cut open, beginning from the pulmonary trunk, and moving peripherally towards segmental and subsegmental arteries, to determine the size and location of the thrombus released from the vein. After removal of the thrombus with tweezers, the part of the PA where the blood clot was previously located was collected for transcriptome analyses. The control animals were first pretreated with atropine and azaperone, and thirty minutes later, propofol and ketamine were given intravenously to induce anesthesia; next, the control pigs were euthanized with sodium pentobarbital (the drugs, their doses and routes of administration were analogous to those used in the experimental group). The entire heart and both lungs were dissected from each animal. Then, very carefully and delicately, PA was exposed in each control animal and cut out for transcriptome analysis (the samples of the PA were collected in a way that exactly corresponded with that applied in the experimental animals, i.e., both the place of sampling and the length of the artery segment were the same). All the animals were kept under standard laboratory conditions. They were fed standard fodder (Grower Plus, Wipasz, Wadąg, Poland) and had free access to water. The animals were housed and treated according to the guidelines of the local Ethics Committee for Animal Experimentation in Olsztyn (affiliated with the National Ethics Committee for Animal Experimentation, Polish Ministry of Science and Higher Education; decision No. 90/2018 of 13.02.2018). All procedures conformed to the guidelines from Directive 2010/63/EU of the European Parliament on the protection of animals used for scientific purposes or the NIH Guide for the Care and Use of Laboratory Animals.

### RNA extraction, library construction and high-throughput sequencing

Total RNA was isolated from the collected parts of the PA of both the experimental and control pigs with the use of the mirVana kit following the manufacturer’s procedure (Thermo Fischer Scientific, Waltham, MA., USA). Extracted RNA was subjected to the quantity and the quality evaluation by the Bioanalyzer 2100 (Agilent Technologies, Waldbronn, Germany). Further, the aliquots with the highest RIN values (RIN > 8) and concentrations (at least 50 ug/ul) were selected for the next steps of sequencing procedures. The sequencing procedure was held by an outsourcing company (Macrogen, South Korea) exploiting Illumina NovaSeq 6000 System (Illumina, San Diego, CA, USA). Briefly, 1 ug of total RNA for each sample was selected for library construction by the Illumina TruSeq mRNA LT Sample Prep kit (Illumina, Inc., San Diego, CA, USA). The laboratory procedure assumed the following steps: the purification of mRNA molecules using poly-T-attached magnetic beads, cutting mRNA into small fragments with divalent cations, amplification of the first-strand cDNA using SuperScript II reverse transcriptase (Invitrogen, Waltham, MA, USA) with random primers and finally the second strand cDNA synthesis with DNA Polymerase I and RNase H. The qPCR Quantification Protocol Guide (KAPA Library Quantification kits for Illumina Sequencing platforms) and TapeStation D1000 ScreenTape system (Agilent Technologies, Waldbronn, Germany) were used to quantify and qualify the RNA-seq libraries. Next, the prepared libraries were transferred to the NovaSeq 6000 platform (Illumina, San Diego, CA, USA), and the sequencing procedure was run with the expected configuration: 150 bp sequence length of paired-end reads and assumed sequencing depth of 40 M reads per sample.

### In silico profiling of pulmonary arteries transcriptome affected by embolism

**Fig. 2:**
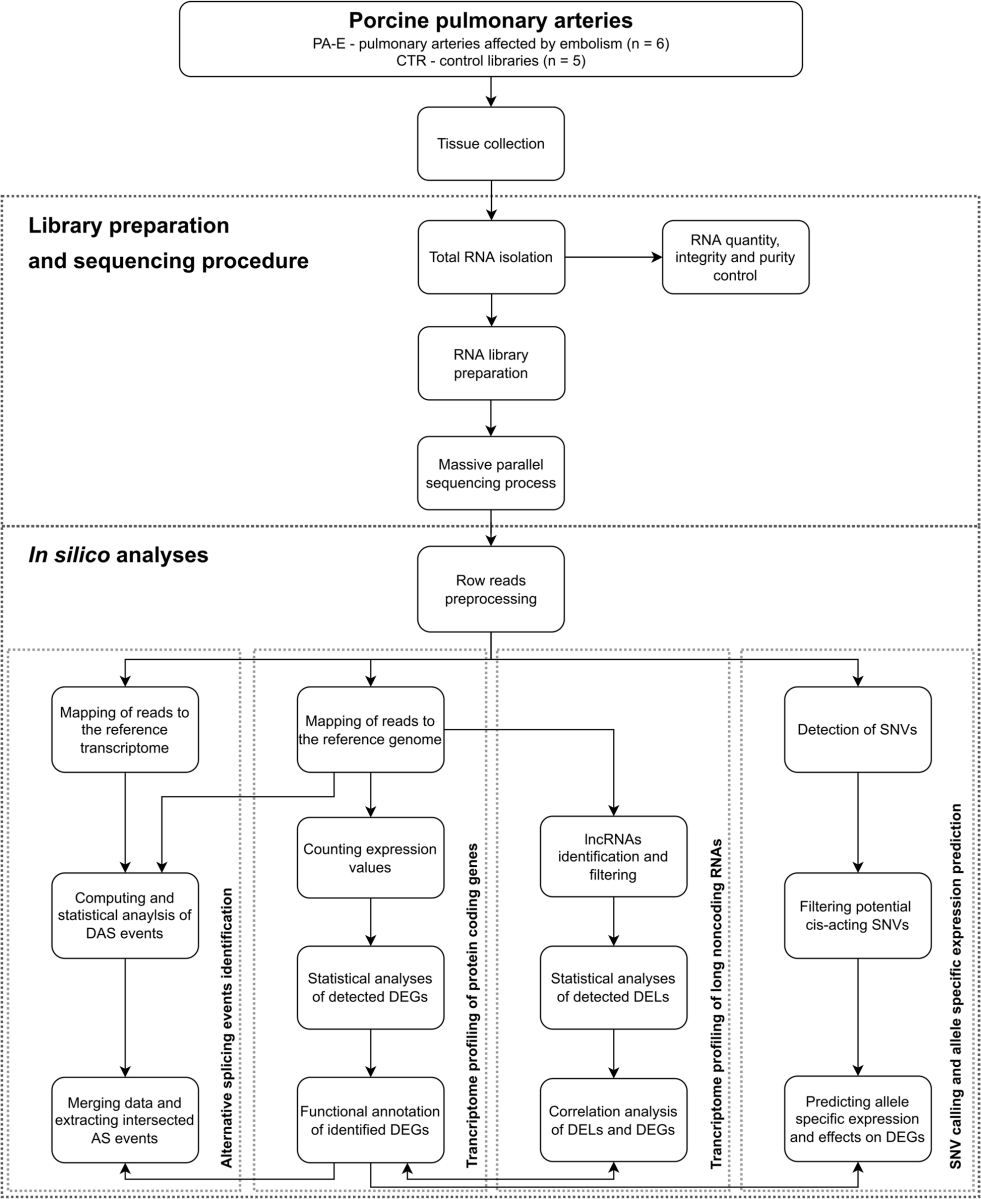
Target enrichment workflow of the experiment assigning step-by-step molecular and bioinformatic procedures. ASEs – allele-specific expression variants; DAS – differential alternative splicing; DEGs – differentially expressed genes; DELs – differentially expressed lncRNAs; lncRNAs – long noncoding RNAs; SNVs – single nucleotide variants

The raw high-throughput sequencing dataset retrieved from NovaSeq 6000 was evaluated according to the quality control standards using FASTQC software version 0.11.7 [26]. The paired-end reads (2 × 150 bp, type stranded) were trimmed after the Illumina adaptors identification within the sequences and also low-quality reads (PHRED cut-off score < 20) were excluded from downstream analysis using Trimmomatic software v. 0.38 [27]. The 120 bp trimmed reads were mapped to the pig reference genome according to ENSEMBL annotation (Sus_scrofa.Sscrofa11.1.99). The mapping results were saved and sorted by coordinate in Binary Alignment Map (BAM) files. Sequences aligned multiple times were not considered for subsequent analysis. The StringTie v. 2.2.1 pipeline [28] was incorporated to re-evaluate the ENSEMBL annotations and BAM files to obtain the novel annotations of intergenic-expressed regions. The integrity of the RNA-seq libraries was validated and clustered with the ggplot2 Bioconductor package v. 3.3.5 of R software v. 4.1.3 (R Core Team; www.R-project.org). Whole-transcriptome high-throughput sequencing (RNA-seq) of PA-E libraries was applied to identify expression profiles of differentially expressed genes (DEGs), other noncoding transcripts, the alternative splicing events and RNA nucleotide polymorphisms (Fig. 2).

### Differentially expressed genes and differentially expressed long non-coding RNA connected with the pulmonary embolism

The differentially expressed analyses were performed for the protein coding and long noncoding RNA (lncRNA) transcripts. The expressed transcripts were grouped according to the genomic localization and tagged as transcriptomic active regions (TARs). The differentially expressed analysis was performed by the binominal statistical test implemented in the DESeq2 method [29]. The TARs localizated in the range of protein coding genes were classified and signed as DEGs if their expression fluctuation between PA-E and CTR was statistically significant (adjusted *p*-value < 0.05). Moreover, lncRNAs with changed expression profiles between compared groups were classified as DELs. Differential expression analysis was conducted on the genomic level. Only long noncoding RNA sequence identification was operated on the transcriptomic level. The research assumed the prediction of all differentially expressed long non-coding RNA (DELs) shinned during the course of PE. The process of lncRNAs identification assumed the prediction of long (> 200 bp) transcripts without coding potential. Noncoding genes with the multi-exonic structures without Rfam and Pfam annotations and characterized by a lack of coding potential, confirmed by three methods—Coding-Potential Assessment Tool (CPAT) [30], Coding Potential Calculator (CPC2) [31] and Flexible Extraction of LncRNAs (FEELnc) [32], were named as lncRNAs. The count gene expression matrix was created from StringTie [28] and Ballgown [33] tools compilation supported by prepDE Python script provided by the StringTie’s authors (https://github.com/gpertea/stringtie/blob/master/prepDE.py; accessed on 21 January 2022). The candidate DEGs and DELs were processed in the MA, volcano, heatmap plots with ggplot2 [34] and circlize R packages v. 0.4.14. *Trans*–regulatory elements were investigated to uncover the relationship between lncRNAs and protein-coding genes. Based on correlations (Pearson’s coefficient—*r*) of lncRNA-mRNA expression profiles, the regulatory connections were statistically confirmed (|*r*|> 0.9) using Hmisc v. 4.7.0 package R software.

### Differential alternative splicing genes involved in pulmonary embolism

Alternative splice junction positions of genes were recovered by the rMATS [35]. Briefly, aligned paired-end reads were evaluated according to the difference in transcript alternative splicing usage profile. The splicing patterns of genes were determined by means of reads arranged around splicing junction sites. The percentage of splicing inclusions (PSI) value was used as a basic unit for splicing event descriptions. Differential alternative splicing genes (DASGs) were statistically tested with the following sensitive parameters: adjusted *p*-value < 0.01 and |ΔPSI|> 0.1. All discovered differential alternative splicing (DAS) events were grouped into five categories: alternative 5’ splice site (A5SS), alternative 3’ splice site (A3SS), mutually exclusive exons (MXE), retained intron (RI) and skipping exon (SE). Significant DAS events were visualized using the maser R package v. 1.12.1 [36, 37] and ggsashimi Python tool v. 0.5.0 [38].

### Single nucleotide variants manifest allele frequency bias

SNVs calling results were achieved by compilation of GATK [39], rMATS-DVR [40] and custom R scripts. After recalibration of BAM files by Picard tool v. 2.6.0 (https://broadinstitute.github.io/picard/; accessed on 13 September 2021), the SNVs were recognized and filtered out according to standard GATK parameters. The applied threshold to filter the SNVs was established on minimum coverage (10 reads). The Variant Call Format (VCF) file was created and transferred to the R environment for the subsequent stages of the filtration procedure. The polymorphic sites were positioned on chromosomes and SNVs ranged within bidirectional transcription regions, intronic flanking regions, simple sequence repeat regions, and pseudogenes regions were eliminated from the further statistical comparison of allele frequency. To elucidate the above filtration procedure the GMATo v. 1.2 [41], BLAT v. 36 [42], BEDTools v. 2.30 [43] tools and vcfR v. 1.12 R package [44] were adapted. The significant allele frequency differentiation (|ΔAAF|> 0.3; FDR < 0.001) was retrieved by counting and comparing reference and alternative nucleotides using Samtools’ *mpileup* function v. 1.6 [45]. The obtained significant results were divided into two groups: localised within genic regions or signed as single nucleotide polymorphism (SNPs) and intergenic or other noncoding SNVs. Both pools of SNVs were tested by VEP [46] to predict the gene structure annotation and effects of polymorphic variants. Based on the smallest standard deviation between biological replicates, the final set of nucleotide substitutions was retrieved and signed as allele-specific expression (ASE). The circos software package [47] was implemented to visualise all variants.

### Functional annotations (gene ontology and KEGG enrichment) of differentially expessed genes, differentially alternative splicing genes and allele-specific expressions

Obtained DEGs, DASGs and ASEs were scanned by gene ontology (GO) and Kyoto encyclopedia of genes and genomes (KEGG) annotation using g:Profiler software [48]. The essential genes were annotated to ontological terms within three aspects, such as biological processes (BP), cellular components (CC), molecular functions (MF) and also assigned to signalling or metabolic pathways (KEGG database) [49]. The enrichment analysis (Fisher's exact test with Benjamini and Hochberg correction; FDR < 0.05) was applied to uncover ontology and pathway annotations regulated by expressed genes and post-transcription mechanisms, including spliceosome and SNV modifications. To obtain the cardiovascular gene signatures, the DEGs were also scanned according to the Human Phenotype Ontology (HP) database according to the terms representing individual phenotypic anomalies (https://hpo.jax.org/app/; accessed on 20 September 2021). To visualise obtained molecular network interaction, a Pathview v. 1.30.1 R package [36] was applied.

### Real-Time PCR validation

The mRNA level of selected genes was determined by Real-Time PCR. The primers for chosen genes were designed using the Primer3Plus software [50] based on the sequences listed in Table S1. The cDNA was obtained with the use of the Applied Biosystems High-Capacity cDNA Reverse Transcription Kit (Thermo Fisher Scientific, Vilnius, Lithuania) according to the manufacturer’s protocol. The Real-Time PCR was performed with the use of Applied Biosystems PowerUp SYBR Green Master Mix (Thermo Fisher Scientific, Vilnius, Lithuania) according to the manufacturer’s protocol on the QuantStudio 3 Real-Time PCR System (Applied Biosystems, Thermo Fisher Scientific Inc., Waltham, MA, USA). In brief, each reaction contained 5 μL of master mix (2X), forward and reverse primers in the amount of 1000 nM of each, 10 ng of cDNA, and a proper volume of nuclease-free water to a final volume of 10 μL. The reactions were performed in four technical replicates for each biological sample. The expression of each gene was calculated using the comparative Pfaffl method [51], where the expression is presented as the fold change relative to the control, as well as normalized to an endogenous reference gene (*ACTB*, GenBank U07786.1, and *GAPDH*, GenBank AF017079) (relative quantification RQ = 1). The results were expressed as means of biological replicates ± standard deviations. Statistical analysis was performed using Student's t-test (two-tailed) in Prism 8 software (GraphPad Software Inc., San Diego, CA, USA). *P*-values were considered statistically significant where 0.0332 (*), 0.0021 (**), 0.0002 (***), and < 0.0001 (****).

## Results

### Pulmonary arteries transcriptomic statistics and the abundance of expression profiles

After sequencing 395 647 360 raw reads were obtained, globally (Table 1). The filtration procedure removed 32 564 994 reads with a poor quality score (PHRED33 < 20). The surviving 363 082 366 paired-end reads were mapped to the porcine reference genome. Results of the mapping process were applied to the identification of the DEGs and DAS events. Uniquely mapped reads contained an average of 96% out of all processed sequences. According to the gene structure, 46.04% of paired-end reads’ nucleotides were aligned to the coding sequence (CDS), 11.24% to the intronic sequences, 26.58% to the untranslated regions (UTR) and 16.14% to the intergenic localizations. The transcriptomic data from this study have been submitted to the European Nucleotide Archive under accession No. PRJEB43020 (https://www.ebi.ac.uk/ena/browser/view/PRJEB43020).

**Table 1:** The statistical metrics for the RNA libraries. The sequencing and mapping results for the 11 RNA-seq libraries: CTR (1–5) refers to the controls; PA-E (1–6) refers to the pulmonary arteries affected by embolism. The uniquely-mapped values were related to the reads mapped to only one location of the porcine genome. The multi-mapped values were assigned to reads aligned to more than one locus on the reference genome. The raw, trimmed and mapped values denote in millions

	**CTR1**	**CTR2**	**CTR3**	**CTR4**	**CTR5**	**PA-E1**	**PA-E2**	**PA-E3**	**PA-E4**	**PA-E5**	**PA-E6**
Raw reads	62.53	65.97	75.22	63.21	65.61	81.30	81.01	71.96	73.29	70.29	80.90
Trimmed reads	58.80	59.76	69.55	56.96	60.07	74.05	74.91	65.52	68.14	64.39	74.00
Percentage of trimmed reads	94.05%	90.59%	92.47%	90.11%	91.55%	91.08%	92.47%	91.05%	92.97%	91.61%	91.48%
Mapped	57.80	58.96	68.74	56.38	59.49	73.16	74.16	64.88	67.37	63.71	73.18
Uniquely mapped	56.16	57.52	66.81	55.16	58.16	71.17	72.30	63.30	65.76	62.23	71.33
Percentage of uniquely mapped reads	97.16%	97.57%	97.19%	97.82%	97.78%	97.27%	97.49%	97.58%	97.62%	97.68%	97.47%

**Fig. 3:**
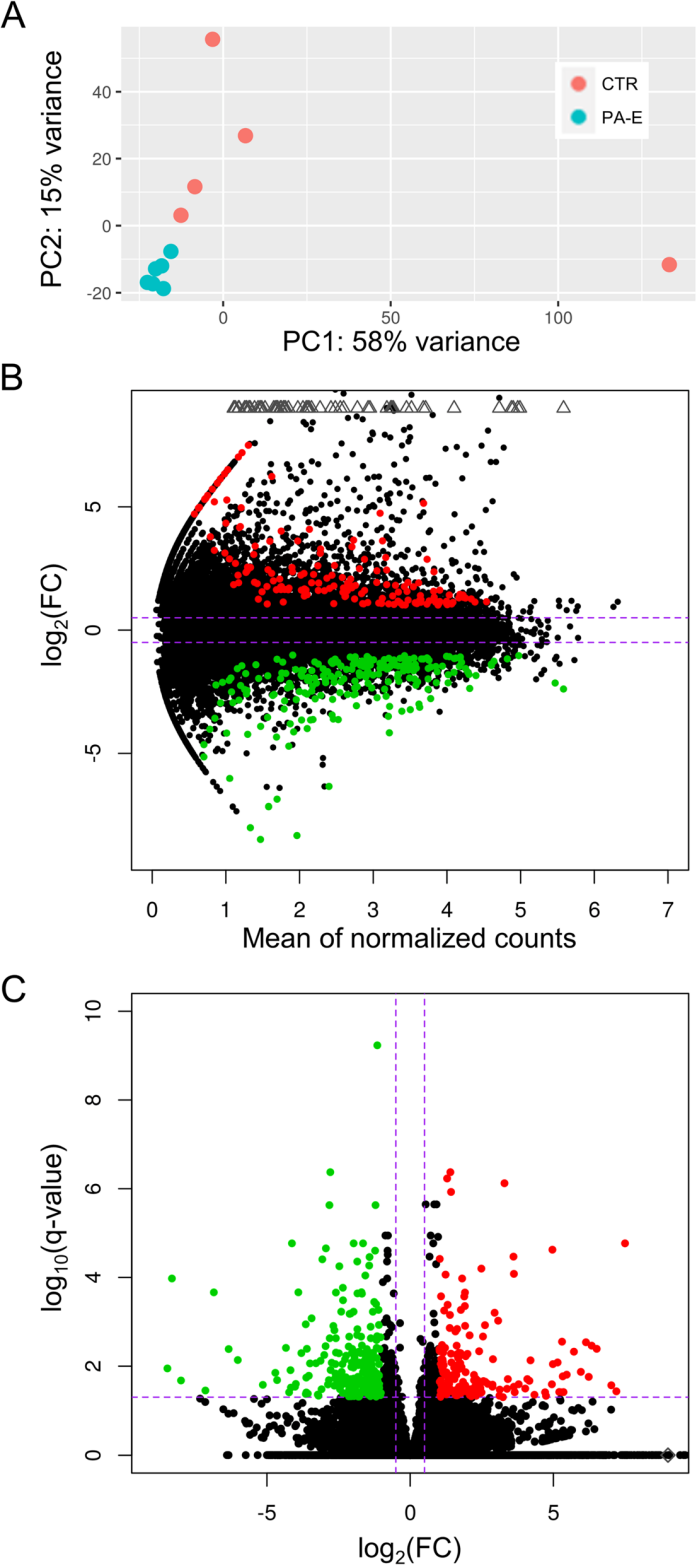
Expression profiles overview of pulmonary embolism. **A** Graphical representation of the first (PC1) and second (PC2) principal components distributed the sample expression pattern of pulmonary arteries affected by embolism (PA-E; *n* = 6) and control (CTR; *n* = 5) libraries. **B** MA chart with logarithmic values of fold change (logFC; Y axis) plotted against normalised counts (X axis) for PA-E and CTR libraries. **C** Volcano plot shows log2FC plotted against negative logarithmic adjusted *p*-values. The dotted horizontal line indicates a negative logarithmic adjusted *p*-value (0.05) cut-off. (**B**, **C**) Red dots represent upregulated differentially expressed transcribed active regions (TARs); green dots depict downregulated TARs; black dots are not significant transcripts, according to DESeq2 method

The principal component analysis (PCA) revealed high homogeneity of expression profiles within the PA-E group. CTR biological replicates indicated less homogeneity (Fig. 3A). Among 33 270 TARs, 3 330 transcripts were highly expressed (FPKM > 100), according to the FPKM sum of all RNA libraries. The 11 923 TARs indicated medium expression (FPKM > 10) and the rest of 18 017 TARs revealed low expression values in PA-E libraries.

### Genes and long noncoding RNAs changed their expression profiles during pulmonary embolism

**Fig. 4:**
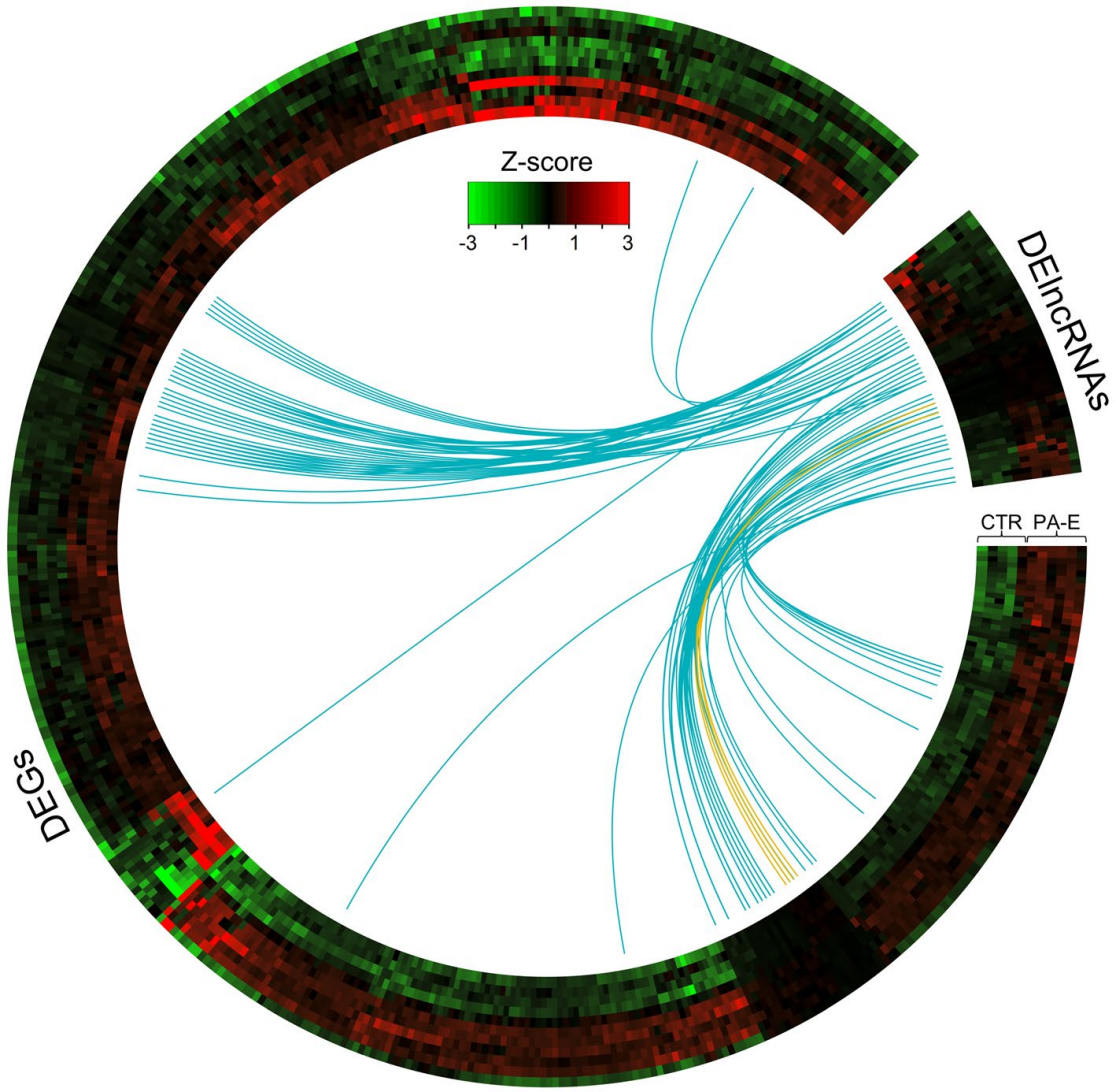
Circular heatmap visualisation of differentially expressed genes (DEGs) and long non-coding RNAs (DELs) in the course of the pulmonary embolism. The 11 upper tracks visualise the normalised (Z-score; red-green scale) expressions for DEGs in each biological replicate (PA-E—pulmonary arteries affected by embolism and CTR-control libraries). The large part of the circle depicts DEGs and the smaller part describes DELs. The inner track shows the correlation links between the co-expressed DEGs and DELs, whereas blue links depict positive (> 0.9) and yellow negative (< -0.9) Euclidean correlation

Screening RNA-seq data for differential gene expression analysis revealed that the PE was associated with 604 differentially expressed TARs (Fig. 3B,C). Among them, 473 TARs were classified as DEGs, which encoded protein *sequences* (Fig. 4*;* Table S2)*.* The depth transcriptome analysis also revealed 46 DELs (Fig. 4; Table S3). *W*ithin DELs, 4 were annotated in the ENSEMBL database and the other 42 were newly-identified in this research. The *trans-*correlation of 32 DELs mediated regulation of 57 DEGs. Most *trans* gene-lncRNA interactions (101) were positively correlated, though only 4 displayed negative expression co-action.

### Alternatively spliced transcripts engaged in pulmonary embolism

**Fig. 5:**
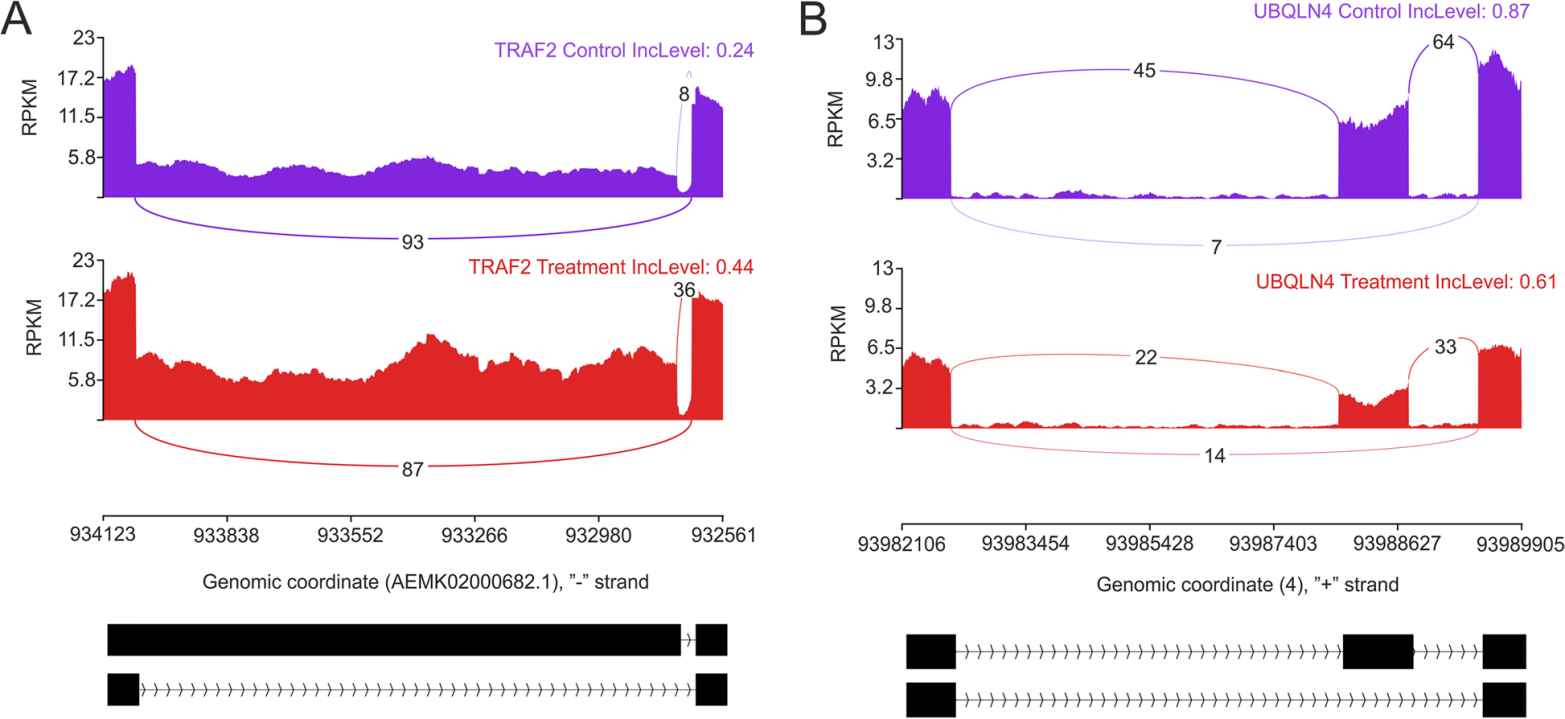
Quantitative visualisation (Sashimi plot) of alternatively splicing events statistically significant in changes of percentage splicing inclusion (adjusted *p*-value < 0.01 and |ΔPSI|> 0.1) between PA-E (pulmonary arteries affected by embolism; red) and CTR (control; violet) samples. The numbers in each upper right corner of the violet and red tracks present the percent splicing inclusion (PSI) values. Splicing differentiations are covered by the number of reads mapped in the range of junction sites (**A**) *TRAF2* (A5SS—alternative 5’ splice site), (**B**) *UBQLN4* (SE – skipping exon). The bottom black tracks show the genomic localisation of splicing events

The reads mapped in the range of acceptor–donor sites were evaluated according to the alternative splicing variants. The splicing detection algorithm (rMATS) allowed the identification of 136 666 alternative splicing events. The 348 splicing events pointed out significant changes in the percentage of exon/intron inclusions in the PA-E vs. CTR comparison. Significant events were incorporated in 229 DASGs. Within detected DASGs, 13 were also classified as DEGs. Among all of the detected DASGs, 43 were classified as A5SS, 56 as A3SS, 27 as MXE, 65 as RI, and 157 as SE (Table S4). Sashimi plots were prepared to visualise exon inclusion levels of the *TRAF2* and *UBQLN4* (Fig. 5).

### Single nucleotide variants callings with a difference in alternative allele fraction as the signature of pulmonary embolism

**Fig. 6:**
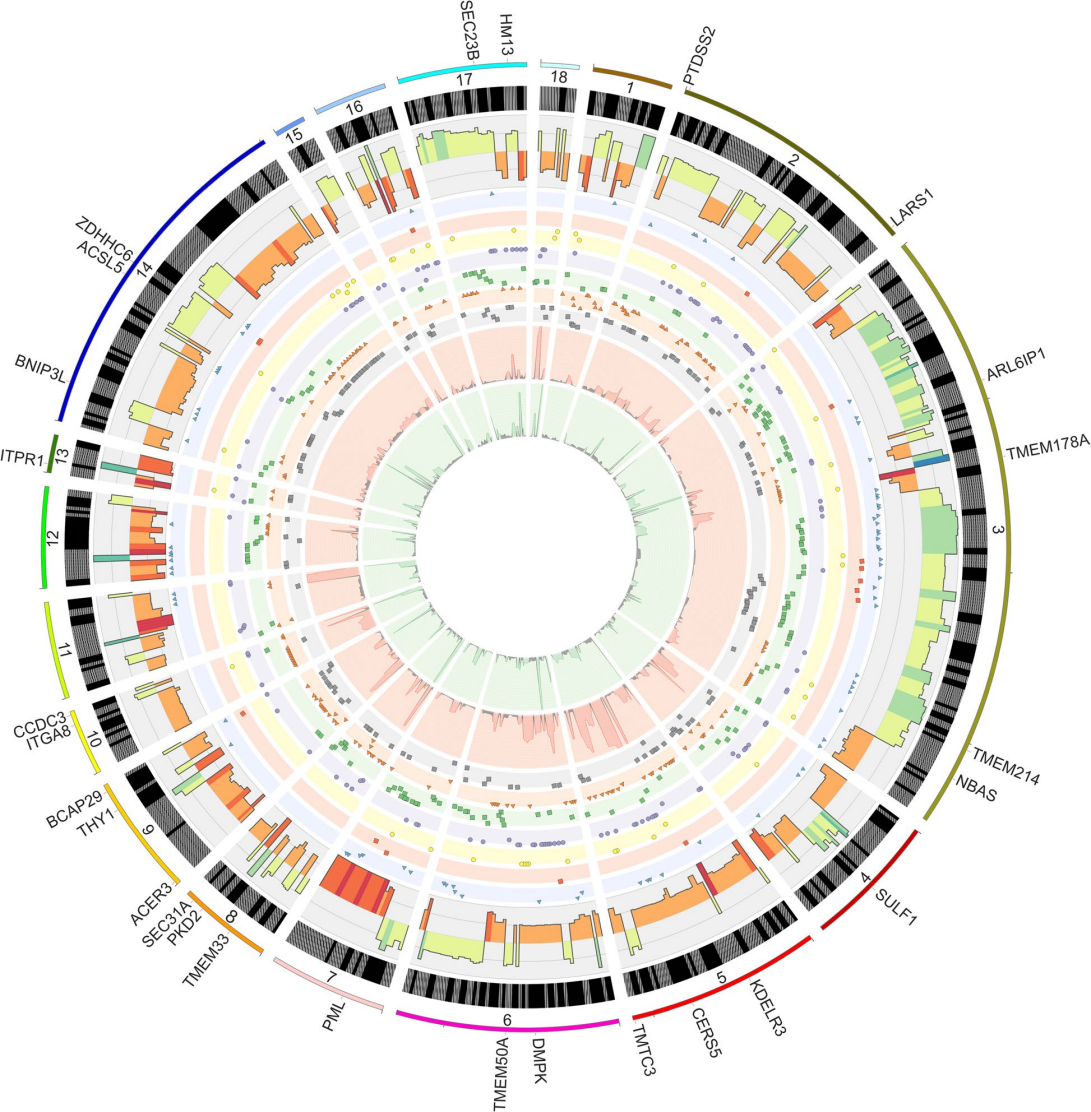
Circos visualisation of single nucleotide variants (SNV) candidates indicated allelic bias between pulmonary arteries affected by embolism (PA-E) and control (CTR) libraries. The external circle illustrates porcine chromosomes and other unclassified scaffolds, where region length is proportional to the number of alternative allele substitutions. The genes belonging to the cellular components of the endoplasmic reticulum were marked on the outer track. The second track depicts canonical (A-G, C-T; black) and other types of nucleotides substitutions (grey). The third track (histogram) depicts differences in the alternative allele fraction (ΔAAF) between PA-E and the CTR libraries. In the eight middle scatter plots, RNA editing substitutions are shown as upstream (blue triangles), 5' UTR (red rectangle), missense (yellow circle), synonymous (purple circle), intron (green squares), splice acceptor (grey circle), 3' UTR (orange triangle) and downstream (grey rectangle). Each inner track shows the coverage of alternative (red histogram) and reference variants (green histogram) in all RNA-seq libraries

In silico investigation revealed the existence of the 456 993 SNVs. To elucidate the SNVs with allele frequency bias between PA-E and CTR samples, the filtration process qualified 70 349 nucleotide variants. The 11 115 ASEs variants revealed statistically significant allelic imbalance (|ΔAAF|> 0.3 and FDR < 0.001) and within them 9 595 were assigned as known porcine SNP candidates (with ENSEMBL ‘*rs*’ ids). Among SNVs, 68.64% of alternative alleles indicated the highest frequencies in the PA-E libraries. The remaining SNPs were annotated giving 9 215 substitutions localised within the protein-coding genes. In accordance with the VEP annotations, 443 SNVs were assigned to lncRNA regions. Identified SNVs were characterised according to all transcripts main annotation regions: upstream (5.78%), 5’ UTR (2.24%), CDS: missense mutations (4.93%) and synonymous mutations (15.71%), introns (29.25%), 3’ UTR (22.48%) and downstream (19.62%). According to the standard deviation measure between biological replicates, the 536 SNVs were selected for the final set of ASEs. Genes localization consequences of the substitution with allele-specific bias within the transcripts components were summarised in Fig. 6. In the range of ASEs, 792 annotations were described by VEP (Table S5). The 61 ASEs were functionally assigned to ‘endoplasmic reticulum’.

### Gene ontology classification of the transcripts expressed in pulmonary arteries affected by embolism

**Fig. 7:**
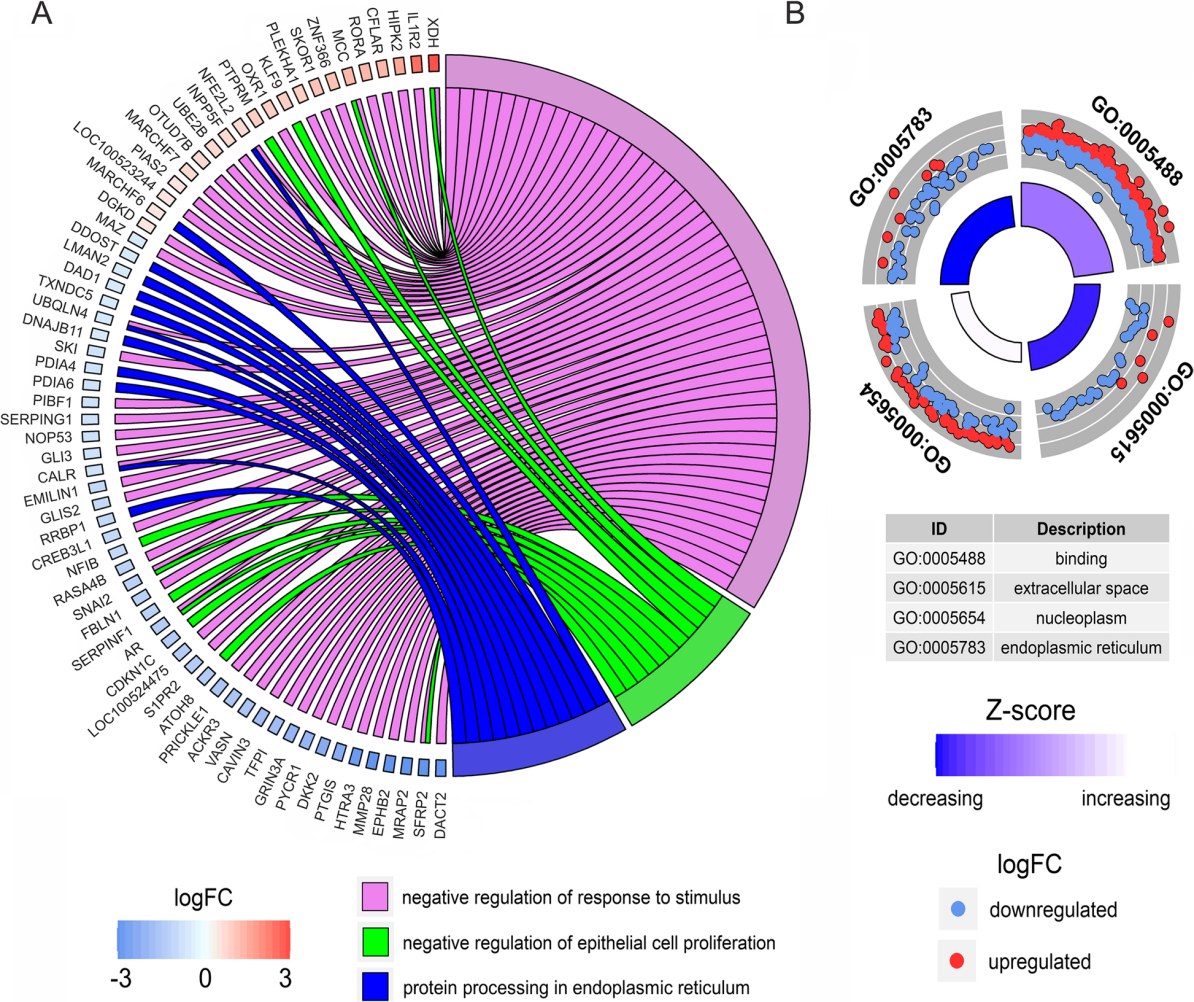
Selected members of the functional and pathway enrichment annotations. **A** Circos plot represents the three most important terms of biological processes (BP) and KEGG pathway associated with differentially expressed genes (DEGs) engaged in pulmonary embolism (PE). Gene symbols with logarithmic values (blue-red scale) of fold change (logFC) are located on the left side of the circos. **B** The outer circle shows the scatter plot for cellular components (CC) and metabolic function (MF) ontology terms enriched with DEGs. Red points represent up-regulated genes and blue points symbolise down-regulated genes. The inner circle contains information on the abundance of genes assigned to the Gene Ontology (GO) term and the overall impact (increasing or decreasing) trend on this functional term. Z-score is calculated using the summarized expression DEGs’ values within particular GO terms

**Fig. 8:**
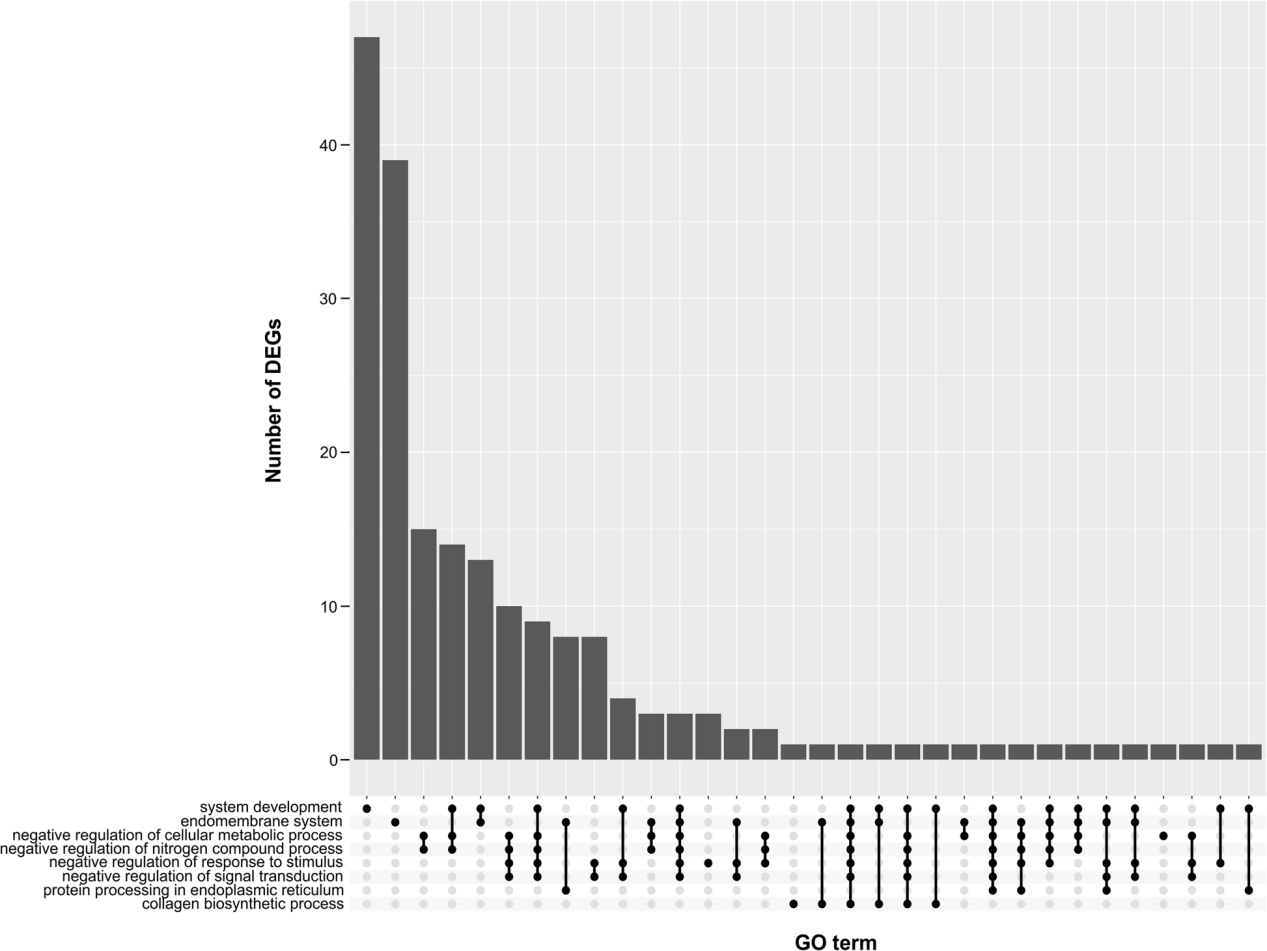
UpSet plot merging most important GO terms and associated DEGs. The heights of the bars indicate the number of DEGs annotated to single or multiple GO terms. Joined dots depict connections between particular GO terms and intersected common DEGs

Active genes involved in PE were classified by GO and 347 of them were assigned to the 27 BP, 324 to the 11 CC and 346 to the 2 MF terms annotations. According to the GO annotation of these genes, the BP enrichment was found for example in the ‘negative regulation of response to stimulus’ and ‘negative regulation of epithelial cell proliferation’ (Figs. 7A and 8).

In the MF category, the DEGs were involved in only two processes of the general meaning, like ‘binding’ and ‘binding protein’ (Fig. 7B). While the CC category grouping revealed that PA-E mRNAs were involved for instance in ‘endoplasmic reticulum’, ‘extracellular space’ and ‘nucleoplasm’ (Fig. 7B). The comprehensive GO enrichment classification was summarised in Table S6.

**Fig. 9:**
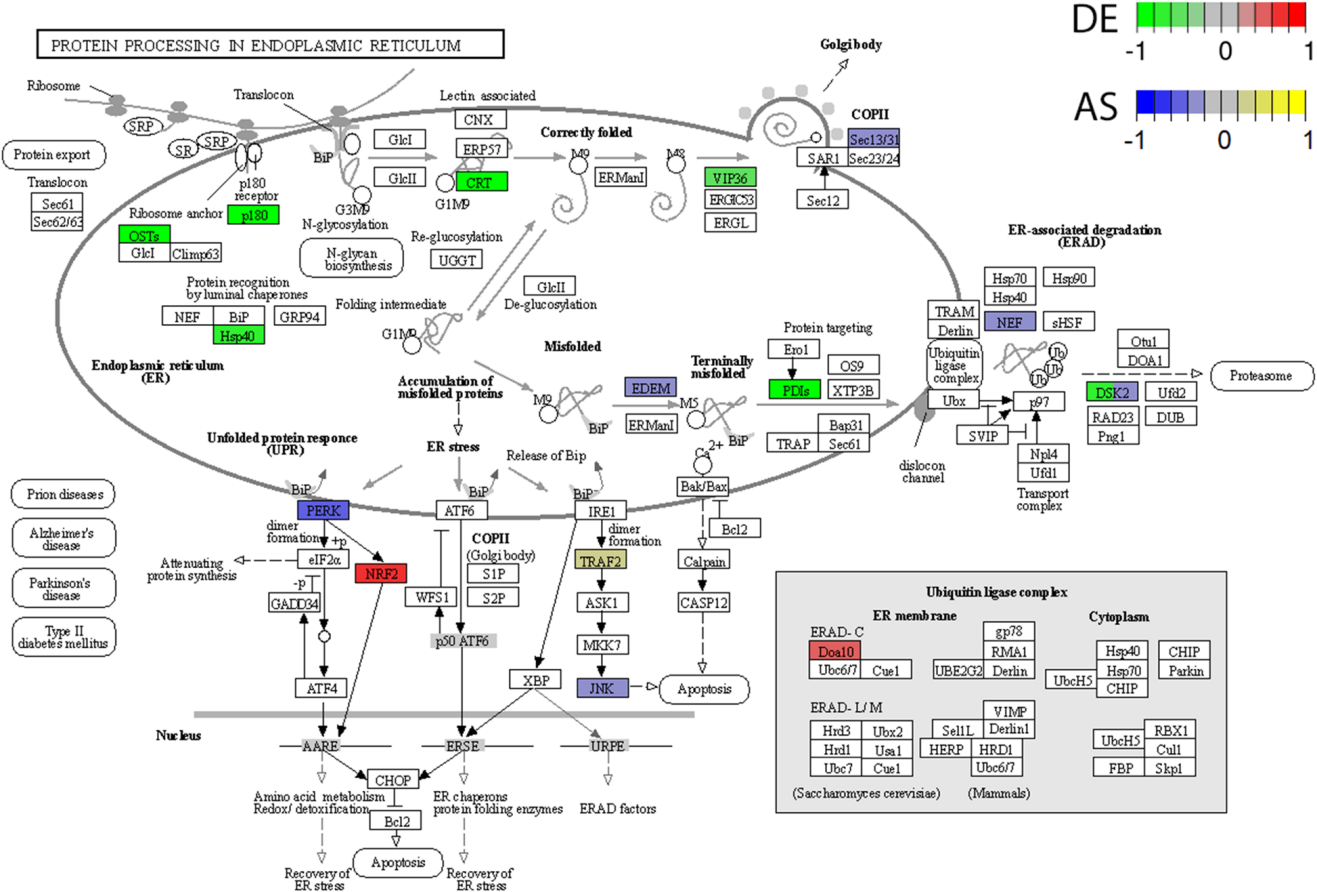
Enrichment Kyoto Encyclopedia of Genes and Genomes (KEGG) analysis of differentially expressed (DE) genes and differentially alternatively splicing (AS) genes engaged in the ‘protein processing in endoplasmic reticulum’. Green and red rectangles present downregulated and upregulated genes, respectively. Blue and yellow rectangles reflect DAS genes with increased and decreased PSI values in pulmonary arteries affected by embolism (PA-E) samples. Logarithmic fold change (logFC) red-green scale describes gene expression values. The PSI purple-yellow scale (|ΔPSI|> 0.1) describes percentage inclusion values

Twelve genes (*DAD1*, *DDOST*, *UBQLN4*, *PDIA6*, *DNAJB11*, *CALR*, *LMAN2*, *PDIA4*, *NFE2L2*, *MARCHF6*, *RRBP1*, *TXNDC5*) belonging to ‘protein processing in endoplasmic reticulum pathway’ were also associated with other GO terms. Eight of those genes were associated directly with the ‘endomembrane system’. Other genes like *CALR*, *NFE2L2*, *UBQLN4* were connected with multiple GO terms, namely: ‘system development’; ‘endomembrane system’; ‘negative regulation of cellular metabolic processes’; ‘negative regulation of nitrogen compound metabolic process’, negative regulation of response to stimulus’ and negative regulation of signal transduction’. Exploring the HP and GO database, DEGs were also grouped according to the potential connection with the development and abnormality of the cardiovascular system. The following DEGs: *AMHR2*, *PTPRB, EPHB2, THBS2, RORA, PTGIS, TMEM204, XDH, EMILIN1, SFRP2, CREB3L1, COL1A2, ACKR3, GLI3, HOXB3, SERPINF1, PIK3C2A, HIPK2, PTPRM, PRICKLE1, COL1A1, GADD45A, FOXC1* were classified as signatures of the ‘cardiovascular system development’ in the course of PE. ‘Abnormality of the cardiovascular system’ was represented by the following DEGs: *AAAS*, *C4A*, *EPHB2*, *GREB1L*, *USP45*, *LIPC*, *BNC2*, *COQ4*, *RBCK1*, *PTGIS*, *WIPI2*, *GUSB*, *TTC7A*, *GUCY1A1*, *SMARCB1*, *ACSL4*, *SERPING1*, *WT1*, *ABCC8, CALR, CRLF1, CEP120, CEP164, COL1A2, BIN1, NFE2L2, IMPDH1, GLI3, LIFR*, *PSMD12*, *MEOX1*, *COL6A1*, *RAD51C*, *EVC2*, *PIBF1*, *CDK4*, *PIK3C2A*, *GGCX*, *COL6A2*, *SELENOI*, *IMPG2*, *ZNF469*, *PTCH2*, *EVC*, *PYCR1*, *GDF6*, *COL1A1*, *FANCF*, *FKBP14*, *NSMCE2*, *TRIM28*, *SNAI2*, *FOXC1*, *CDKN1C*, *SKI*, which may be crucial as genetic biomarkers of the PE etiology. In the signaling pathway analysis, one KEGG pathway was identified for the mRNAs modulated during PE. Within twelve genes enriched in KEGG ‘protein processing in endoplasmic reticulum’ pathway, ten were underexpressed (*DAD1*, *DDOST*, *UBQLN4*, *PDIA6*, *DNAJB11*, *CALR*, *LMAN2*, *PDIA4*, *RRBP1*, *TXNDC5*) and two others were overexpressed (*NFE2L2*, *MARCHF6*) (Figs. 7A, 8 and 9*).* The same KEGG pathway (ssc04141) was also exposed by DASGs (*EIF2AK2*, *UBQLN4*, *BAG2*, *SEC31A*, *EDEM2*, *EIF2AK4*, *TRAF2*, *MAPK8*), with alternative splicing bias between PA-E and CTR samples (Fig. 9; Table S7). Within SNVs with significant allelic imbalance, the 61 polymorphisms were localized in the vicinity of the following genes: *ITGA8*, *CCDC3*, *ITPR1*, *BNIP3L*, *ACSL5*, *ZDHHC6*, *SEC23B*, *HM13*, *LARS1*, *PTDSS2*, *TMEM178A*, *TMEM214*, *NBAS*, *ARL6IP1*, *SULF1*, *CERS5*, *TMTC3*, *KDELR3*, *DMPK*, *TMEM50A*, *PML*, *PKD2*, *SEC31A*, *TMEM33*, *BCAP29*, *ACER3*, *THY1*, that belong to the cellular components of ‘endoplasmic reticulum’ (Fig. 6).

Comparison of DEGs detected within this research with DEGs from other studies focused on expression analysis in DVT and PE revealed that some of them were overlapping, regardless the species, model, or transcriptomic assay used (Fig. S1). Among 473 identified DEGs, 29 were present also in analyzed other data sets [14, 52–54]. Most of the DEGs (*IGFBP6*, *MOXD1*, *SLC4A11*, *TTYH3*, *ATOH8*, *PDIA6*, *SLK*, *VCAN*, *ME3*, *COL1A2*, *PSMD12*, *CNP*, *DACT2*, *ENTPD4*, *SHMT2*) were overlapping between current project and data from Tang et al. [14] and Zagorski et al. [52]. There was one DEG uncovered in all of the analyzed datasets—*PLOD2*.

### Gene expression quantification

The genes for validation were selected following the assessment of the function, expression values and read’s distribution within samples. The overexpression of *LIFR* and *PIK3C2A* genes (over 2 arbitrary units compared to the control) was assigned to the abnormalities of the cardiovascular system, making them crucial biomarkers of the PE etiology. The significant overexpression in PA-E compared to the CTR libraries was also noted in the *ACSL4* and *SELENOI* engaged in vascular homeostasis and various membrane-related cellular processes. On the other hand, *COL1A1, COL1A2* and *COL6A1,* which are also crucial for vascular remodelling, were significantly downregulated in PA-E libraries, the same as *C4A* involved in local inflammatory processes. The RNA-Seq results were validated using Real-Time PCR which revealed overexpression of six genes: *ACSL4*, *ENSSSCT00000024889*, *LIFR*, *PIK3C2A*, *SELENOI* and *WDR37* whereas underexpression of another six genes: *C4A*, *COL1A1*, *COL1A2*, *COL6A1*, *PTGIS* and *PYCR1* (Fig. S2).

## Discussion

DVT and PE are common life-threatening conditions, therefore the understanding of their molecular background is of great importance, as the identification of genes and pathways is crucial in disease pathomechanism and may reveal potential treatment targets. The current research, which aimed at analyzing the changes in the transcriptome of PA with induced PE, is the continuation of our previous studies devoted to the transcriptomic profiling of femoral veins in DVT. We mainly disclosed that dysregulation of TNF, NF-κB, and apoptosis pathways affect inflammatory responses and the formation, remodeling, and resolution of thrombus in the course of DVT [16]. Whilst, the present project not only uncovered the genetic biomarkers of PE but also unrevealed that the disturbed homeostasis in the biosynthesis of proteins in the ER plays a major role in the pathogenesis of PE.

### Genetic biomarkers of the pulmonary embolism (PE)

The present research revealed several DEGs involved in vascular wall integrity and strongly associated with dysfunction of the cardiovascular system, which may be potentially considered genetic biomarkers of PE. In particular, underexpression of *COL1A1* (logFC = -2.39), *COL1A2* (logFC = -2.14), *COL6A2* (logFC = -1.17), *COL6A2* (logFC = -1.17), *C4A* (logFC = -1.56), *GLI3* (logFC = -0.89), and overexpression of *ACSL4* (logFC = 1.27), *PIK3C2A* (logFC = 1.02) genes in the PA-E libraries is worth emphasizing. The extracellular matrix, composed of collagens, elastin, and proteoglycans, is an essential component of the PA wall [55]. Our study disclosed four underexpressed DEGs in the PA-E, which were associated with collagen synthesis: Collagen Type I Alpha 1 Chain (*COL1A1*), Collagen Type I Alpha 2 Chain (*COL1A2*), Collagen Type VI Alpha 1 Chain (*COL6A1*) and Collagen Type VI Alpha 2 Chain (*COL6A2*). *COL1A1* and *COL1A2* belong to group I collagen (COL1; fibrillar forming collagen), whereas *COL6A1* and *COL6A2* belong to group VI collagen (COL6), which acts as a cell-binding protein [56]. COL1 is the main type of collagen, present in all three layers of the vessel wall [55], and it not only provides structural support but also is crucial for the growth, homeostasis and repair of the tissue [57]. COL6, which is non-fibrillar collagen, is engaged in the vascular wall remodeling process. It has been found, that *COL6* was significantly decreased in the tunica media of rat PA, following the hypoxia [58]. Thus, it can be concluded that the amount of COL6 has a strong impact on the proliferation and migration of vascular smooth muscle cells. Undoubtedly, the accumulation of the clot in the PA creates hypoxic conditions, which may be the reason for *COL61* and *COL62* underexpression. Intriguingly, Luther et al. [59] revealed that deletion of *COL6* in knockout model mice plays a critical protective role following myocardial infarction by limiting infarct size, chronic apoptosis, aberrant remodeling, and fibrosis. Therefore, the underexpression of *COL* genes observed in the present experiment may potentially reflect protective mechanisms triggered to preserve the functional integrity of the vessel wall. Furthermore, Complement C4A (*C4A*), GLI Family Zinc Finger 3 (*GLI3)* and Acyl-CoA Synthetase Long Chain Family Member 4 (*ACSL4*) genes which were differentially expressed in the investigated samples, have also a strong impact on the vascular integrity. C4A participates in the complement classical activation pathway [60]. Derived from proteolytic degradation of complement C4, C4A anaphylatoxin is a mediator of local inflammatory processes [61]. It induces the contraction of smooth muscles and increases vascular permeability. An increasing body of evidence supports the functional role of complement activation in the pathogenesis of cardiovascular disease through pleiotropic effects on endothelial, hematopoietic cell function, and haemostasis [62]. Therefore, the observed underexpression of *C4A* may again reflect the protective processes induced to limit the local thromboinflammation. Possibly this effect may be enhanced by the underexpression of *GLI3*, which acts as a transcription factor that regulates vessel growth, endothelial-cell activity, and apoptosis [63]. It has been found that overexpression of *GLI3* upregulates the expression of pro-angiogenic factors in endothelial cells, including interleukins and chemokines [63]. Thus, respectively, its downregulation may mute inflammation. Acyl-CoA Synthetase Long Chain Family Member 4 (*ACSL4*) expressed in arterial smooth muscle cells may regulate some processes dependent on the release of arachidonic acid mediators [64]. For instance, it has been found that the overexpression of *ACSL4* results in increased synthesis of eicosanoids that play important roles in vascular homeostasis. Moreover, ACSL4 modulates prostaglandin E2 release from smooth muscle cells [64]. These observations suggest that the upregulation of *ACSL4* disclosed in the PA-E samples, may, again reflect the local inflammation process and elucidates the usefulness and necessity of non-steroidal anti-inflammatory drug application in the PE treatment. Additionally, the present study revealed the overexpression of Phosphatidylinositol-4-Phosphate 3-Kinase Catalytic Subunit (*PIK3C2A*) in the PA-E. Phosphatidylinositol 3–kinases (PI3K) are recruited during the clot formation after binding collagen to GPVI on platelets. Activation of PI3K causes the release of secondary mediators, such as ADP and thromboxane A2, culminating in platelet aggregation mediated by fibrinogen [65]. Many aspects of cardiovascular physiology and disease have been linked to PI3K isoforms. The key roles of PI3Ks in platelets suggest that they could all represent valid antithrombotic targets, to inhibit thrombosis while having a limited effect on normal haemostasis [66].

### Protein processing in the endoplasmic reticulum (ER) pathway

The endoplasmic reticulum (ER) is a large cellular organelle that serves important functions in the cell, including synthesis, folding, modification, and transport of proteins, production of lipids and sterols, as well as storage of free calcium [67]. Numerous cellular stresses can lead to disturbances between the demand for protein folding and the capacity of the ER to efficiently sustain this process, thereby initiating ER stress pathway [68]. This specific pathway is involved in a wide range of pathologies [69], for instance, it may play a crucial role in the development of cardiac hypertrophy and heart failure [70]. ER stress pathway is activated to protect cells, but when stresses are severe, apoptosis is induced to remove damaged units. Current in-depth bioinformatics analysis revealed that the ‘protein processing in endoplasmic reticulum’ pathway (ssc04141; Fig. 8) significantly contributes to the pathogenesis of PE. Our research revealed twelve genes enriched in this KEGG pathway, ten were underexpressed: *DAD1* (logFC = -0.60)*, LMAN2* (logFC = -0.58)*, RRBP1* (logFC = -1.09)*, DDOST* (logFC = -0.59), *PDIA4* (logFC = -0.76)*, UBQLN4* (logFC = -0.70), *DNAJB11* (logFC = -0.71)*, PDIA6* (logFC = -0.77)*, CALR* (logFC = -0.90), *TXNDC5* (logFC = -0.60), and two others were overexpressed: *NFE2L2* (logFC = 0.69), *MARCHF6* (logFC = 0.44). The obtained results indicate that genes within this pathway were not only differentially expressed but also exposed by DASGs (*EDEM2*, *MAPK8*, *TRAF2*, *UBQLN4*, *EIF2AK2*, *BAG2*, *SEC31A*, *EIF2AK4*) and significant allelic imbalance.

Our study reveals, that PE was followed by downregulation of Defender Against Cell Death 1 (*DAD1*), which is one of the genes that contribute to the synthesis of oligosaccharyltransferase (OST) complex that catalyzes the transfer of a high mannose oligosaccharide onto asparagine residues. It has been found that disruptions in the OST complex induce chronic ER stress by activating one of the pathways in unfolded protein response (UPR), leading to caspase-dependent apoptosis [71]. Thus, most possibly, the underexpression of *DAD1* observed in the PA-E libraries represents cell death initiated by severe ER imbalance induced by PE. The downregulation of Lectin Mannose Binding 2 (*LMAN2*) and Ribosome Binding Protein 1 (*RRBP1*) disclosed in the present study, also reveal distinct disruption in ER protein processing. LMAN2 is an intracellular protein that shuttles between the Golgi apparatus and ER, responsible for recognizing misfolded proteins that are produced under the ER stress conditions [72]. In turn, *RRBP1*, underexpressed in PA-E arteries, takes part in polysome assembly and therefore in protein biosynthesis along with the expansion of the Golgi complex. Additionally, *RRBP1* may influence protein synthesis, necessary in some unfolded protein response (UPR) pathways [73]. Another underexpressed gene Dolichyl-Diphosphooligosaccharide Protein Glycosyltransferase (*DDOST;* also known as *AGER1*) is a receptor of advanced glycation end products (AGEs) and it can decrease prooxidant actions [74]. Accumulation of AGEs, resulting from *DDOST* overexpression, triggers platelets hyperreactivity and in specific circumstances may promote thrombosis [75]. Therefore, correspondingly, the observed *DDOST* underexpression most possibly should be considered as a protective mechanism, decreasing platelets activation, thus, leading to blood clot degradation. Disulfide bond formation, a critical step in protein folding which occurs within the ER, is catalyzed by the protein disulfide isomerase family (PDI). One of the PDI members Protein Disulfide Isomerase Family A Member 4 (*PDIA4*) activates platelets and promotes thrombosis initiation [76]. Accumulation of abnormally folded proteins and increased *PDIA4* expression trigger ER response. Additionally, *PDIA4* is highly transcribed in the megakaryocytes and effectively joins with the cell surface following platelet activation [77]. Therefore, most possibly underexpression of *PDIA4* observed in the PA-E samples, similarly to the underexpression of *DDOST* reflects the protective mechanism limiting further clot formation in the affected PA.

So far, there are no reports supporting the direct effect of Ubiquilin 4 (*UBQLN4*) and DnaJ Heat Shock Protein Family (Hsp40) Member B11 (*DNAJB11*) on the course of PE. Both these genes, which are downregulated in PA-E libraries, are associated with cytosolic chaperones, major regulators of ER protein folding and ER stress [78]. Cytosolic chaperone UBQLN4 belongs to the ubiquitin-binding proteins, which exhibit diverse biological functions, including enhancement of autophagy-mediated degradation and participation in ER-associated protein degradation [79]. *DNAJB11* encodes one of the main cofactors of the ER chaperon, a heat-shock protein that performs multifaceted functions in coordinating ER and extracellular proteostasis, required for efficient protein folding and trafficking [80]. Furthermore, Protein Disulfide Isomerase Family A Member 6 (*PDIA6*) is an ER-resident protein that plays a crucial role in blocking the onset of ER stress, as it suppresses the activation of the UPR [81, 82], therefore the observed *UBQLN4, DNAJB11* and *PDIA6* underexpression, again reflects the ongoing ER stress response in the PA-E. Another downregulated gene, calreticulin (*CALR*), is well-known for sequestering calcium within the ER lumen [83]. Mutations within *CALR* have been shown to occur in 67% of essential thrombocythemia (ET) cases. ET is a myeloproliferative neoplasm characterized by a clonal expansion of megakaryocytes, which can result in both arterial and venous thrombosis [84].

Our research revealed two overexpressed genes involved in the ‘protein processing in endoplasmic reticulum’ pathway—NFE2 Like BZIP Transcription Factor 2 (*NFE2L2*) and Membrane Associated Ring-CH-Type Finger 6 (*MARCHF6*)*. NFE2L2* controls cellular response to oxidative insults, thus preventing damage to components sensitive to redox changes such as proteins, lipids and DNA. *NFE2L2* is a putative regulator of the cardiac stress-response [85], and it has been found that *NFE2L2* gene polymorphisms are associated with the risk and severity of acute type A aortic dissection [86]. MARCHF6 is a ubiquitin ligase embedded in the membranes of the ER, which participates in ER-associated degradation, including autoubiquitination [87]. Moreover, MARCHF6 is involved in vascular endothelial-cadherin-based adherens junctions function and sprouting angiogenesis [88].

This project examined also the relationship between several genes within the identified ER-KEGG pathway and genes with alternative splicing bias, exposed by DASGs. For instance, a significant allelic imbalance was detected in ER Degradation Enhancing Alpha-Mannosidase Like Protein 2 (*EDEM2*), Mitogen-Activated Protein Kinase 8 (*MAPK8*; also known as *JNK1* or *JNK*), TNF Receptor Associated Factor 2 (*TRAF2*) or in previously described *UBQLN4* genes. It has been found, that SNPs with the genome-wide significance of the *EDEM2,* influence protein C concentration, which is an important endogenous anticoagulant in hemostasis and its deficiencies significantly increase the risk of VTE [89]. *MAPK8* is a member of the mitogen-activated protein kinase family (MAPK). MAPK signaling pathway plays a vital role in the pathogenesis of cardiovascular diseases, for example, *MAPK8* was identified as the target gene in myocardial infarction [90].

As was already mentioned in the Results section, in the PA-E libraries we have found not only DEGs directly involved in the ‘protein processing in endoplasmic reticulum’ pathway but we have also revealed 61 polymorphisms localized in the vicinity of the genes belonging to the cellular components of ER, for example, *ITGA8*, *CCDC3*, *ITPR1*, *PKD2*, *BNIP3L*, *SEC23B*, *CERS5*, *ACER3*, *KDELR3*, *PML*, *SEC31A*, *THY1* and *BCAP29*. These genes, similar to the previously described DEGs, are entangled in the protein biosynthesis process and ER stress-induced apoptosis.

### TNFα signaling pathway

A previous analysis of the changes in the transcriptome of the femoral vein caused by DVT in the porcine model based on the formation of the thrombus in vivo, disclosed that TNF, NF-κB and apoptosis pathways are strongly dysregulated during the thrombus formation [16]. The present study also revealed genes with significant allelic imbalance involved in the TNFα signaling pathway, such as the Coiled-coil domain containing 3 (CCDC3), Thy-1 Cell Surface Antigen (Thy1) and *TRAF2. CCDC3* is highly expressed in vascular endothelial cells where it is involved in the regulation of TNFα-induced inflammatory response [91]. It has been found, that TNFα, which is responsible for triggering the expression of pro-inflammatory genes by activation of NF-κB, downregulates *CCDC3* expression in endothelial cells [92]. Moreover, Azad et al. [91] have proven that the interaction between *TNFα* and *CCDC3* is bidirectional, and an increased *CCDC3* level inhibits *NF-κB* activation and thus decreased TNFα-induced endothelial gene expression. *Thy1* is expressed in microvascular endothelial cells during angiogenesis [93]. The evidence suggests that Thy-1 induced on the endothelium regulates vascular permeability at sites of inflammation [94]. The expression on endothelial cells**,** the inducibility by TNF, and the ability to transmit signals suggest that *Thy-1* may again have an important role in inflammatory responses [95]. *TRAF2* encodes proteins belonging to the TNF receptor-associated factor (TRAF) protein family, which mediate the transduction of signals from the tumor necrosis factor (TNF) receptor superfamily. This protein is required for TNFα—mediated activation of *MAPK8* and nuclear factor κB (*NF-κB*), for example in response to ER stress. The activation of *MAPK8* is essential for the regulation of TNFα induced apoptosis [96]. Activated *NF-κB* is a factor that modifies the transcription of numerous target genes encoding pro-inflammatory cytokines, chemokines, cell adhesion molecules, and enzymes that produce pro-inflammatory factors, such as nitric oxide or prostaglandins.

#### The comparison of the present data with the results obtained in other animal and human models

To gain a deeper insight into the molecular background of pathomechanisms that lead to PE currently detected DEGs were compared with the results of four other research performed either in humans or in other animal models (Fig. S1). These studies included: an analysis of the human VTE patients’ microarray dataset [53], an investigation of the differential effect of pulmonary vascular occlusion during mild and severe PE on the lung transcriptome in the rat [52], data mining of public gene expression datasets from VTE and other cardiovascular diseases (i.e. myocardial infarction, peripheral arterial occlusive disease and stroke; [54]) and genome-wide gene expression profiling in rabbit autologous thrombus PE model [14]. Intriguingly, we have found twenty-nine overlapping genes which were also differentially expressed in the present study. Four of these DEGs (*ACSL4*, *FKBP14*, *PDIA6*, *COL1A2*), were already mentioned in the Discussion section. However, the most striking observation to emerge from the data comparison was that Procollagen-Lysine,2-Oxoglutarate 5-Dioxygenase 2 (*PLOD2*) gene was uncovered in all of the analyzed datasets, which may indicate its crucial role in PE pathogenesis. PLOD2 protein is involved in the stability of collagen fibrils, that in turn plays a key role in the maintenance of integrity and elasticity of normal as well as atherosclerotic vessel walls and it stimulates the thrombus formation processes [97]. Although compared DEGs were associated with distinct pathogenic mechanisms obtained data suggest that the mechanisms responsible for cardiovascular diseases such as PE and VTE are at least partially overlapping. Thus, such interspecies comparison, including results from various methodological approaches may constitute the fundamental framework for future clinical research on human cardiovascular diseases, hopefully leading to the identification of potential biomarkers for prognosis or therapeutic targets.

### Potential limitations

Like all experimental models, ours has some limitations that must be recognized. Unlike humans, animal species do not develop spontaneous VTE, therefore despite the fact that pigs share many similarities with humans, the creation of thrombus in their veins requires the application of external factors. Thus, it is difficult to entirely relate also PE pathomechanism in pig to man. However, it must be stressed that this problem refers to all VTE animal models.

## Conclusions

This research provides the first thorough investigation of the changes in the genes expression profile and other posttranscriptional modifications affected by the blood clot lodged in the PA in the course of PE. The current data revealed that dysregulation of protein biosynthesis in ER plays a crucial role in PE pathogenesis. The differentially expressed genes associated with the examined ER pathway may exert a twofold impact on the course of PE, resulting from both their main function and being a link between disturbed ‘protein processing in endoplasmic reticulum’ pathway and ER stress response induced by the clot. Downregulated genes were mostly involved in ER primary function, that is protein synthesis and maturation (including their cleavage, glycosylation, folding and assembly). Whereas, the upregulated genes were involved in the regulation of the redox status, sterol, and lipid metabolism. Furthermore, the present study revealed DEGs classified as signatures of the dysfunction and abnormality of the cardiovascular system. The findings of this research provide insights for understanding PE pathogenesis and its complications. Therefore, we hope that our research will lay the groundwork for future clinical trials investigating new therapeutic agents.

## Supplementary Information

**Additional file 1: Table S1.** Sequences of primers used in Real-Time PCR validation for differentially expressed protein-coding genes and reference genes.

**Additional file 2: Table S2.** Differentially expressed protein-coding genes identified in the porcine pulmonary arteries affected by embolism.

**Additional file 3: Table S3.** Differentially expressed long noncoding RNAs identified in the porcine pulmonary arteries affected by embolism.

**Additional file 4: Table S4.** Differentially alternative splicing events discovered by rMATS tools. Five alternative splicing event types show an imbalance in the percent splicing inclusion (PSI) values between CTR and PA-E libraries.

**Additional file 5: Table S5.** Differences in alternative allele fraction for significant allele-specific expression sites identified in the porcine pulmonary arteries affected by embolism.

**Additional file 6: Table S6.** Differentially expressed genes enriched in the Gene Ontology annotations.

**Additional file 7: Table S7.** Differentially alternative splicing genes classified to the Gene Ontology terms.

**Additional file 8: Figure S1.** Venn diagram of DEGs comparing the obtained results with other datasets focusing on expression analysis of the DVT and PE. The numbers within the intersected ovals indicate the count of DEGs common within experiments. The outer ovals depict DEGs which are unique in the particular research.

**Additional file 9: Figure S2.** The mRNA expression of selected genes obtained using Real-Time PCR. The expression of endogenous controls is shown as normalised to a value 1, and the samples indicate the changes relative to the controls. All the relative expression units [a. u.] were presented as logarithmic values. The exact values of expression are above the bars. *P*-values were considered statistically significant where 0.0332 (*), 0.0021 (**) and <0.0001 (****); *ns* - not significant, but tended to have expression changes.

## Data Availability

Data relating to this article can be found in the article itself or online supplementary material. Raw data were submitted to the European Nucleotide Archive (ENA; (https://www.ebi.ac.uk/ena/browser/view/PRJEB43020) under accession No. PRJEB43020 and used for transcriptome profiling.
