# Supplementary material for: Pulmonary artery embolism: comprehensive transcriptomic analysis in understanding the pathogenic mechanisms of the disease
Source: BMC Genomics. 2023 Jan 9;24:10. doi: 10.1186/s12864-023-09110-0 (PMC9830730; doi:10.1186/s12864-023-09110-0)
Supplement: Supplementary file 1 — Additional file 1: Table S1. Sequences of primers used in Real-Time PCR validation for differentially expressed protein-coding genes and reference genes. [file 12864_2023_9110_MOESM1_ESM.docx]

Table S1. Sequences of primers used in Real-Time PCR validation for differentially expressed protein-coding genes and reference genes.

| Name | Forward sequence: (5' to 3') | Reverse sequence: (5' to 3') |
| --- | --- | --- |
| *WDR37* | ACGTGGGGTCAGTGAATTCC | GTCAGGCTCGTCCTTATCGG |
| *SELENOI* | CCAACATCACATGCCAGCTG | AATGGATGTGGGCCAGAGTG |
| *PYCR1* | GGAAGATGGGGGTGAACCTG | CATGCAGCGGATGACTTTGG |
| *PTGIS* | ATCTTTACCGTGCTGGTGGG | CAGCTGCAGTTCTCTGTGGA |
| *PIK3C2A* | CAAAATCTGGCCCGAACAGC | GGTCTACTGCTCTGTGCTGG |
| *LIFR* | ATACAGACGGAGGAATGGGC | AAGAGCACTGTTTCCCTCACA |
| *ENSSSCT00000024889* | CGGGCATCTACAGTGGTGAA | TCCAAGGCCTTTTATCGGGC |
| *COL6A1* | AACGGCACTAAAGGCTACCC | ACTCACAGCAGGAGCACATC |
| *COL1A2* | CGAACCTGGTCAGACTGGTC | CCCTAATGCCCTTGAAGCCA |
| *COL1A1* | AAGTTTGAGCCTGGGCAGTC | ATCCGGTTGTTCTGAGTGG |
| *C4A* | CCTCAACATGGCCAAGGTCT | AGAGTACCGGCCCAGAATCT |
| *ACSL4* | CTGGACTGGGACCAAAGGAC | CCATGATTTCCGGGACAGCT |
| *ACTB* | GGATGCAGAAGGAGATCACG | ATCTGCTGGAAGGTGGACAG |
| *GAPDH* | CACCATCTTCCAGGAGCGAG | GGTTCACGCCCATCACAAAC |
