# Supplementary figures and images for: Pulmonary artery embolism: comprehensive transcriptomic analysis in understanding the pathogenic mechanisms of the disease
Source: BMC Genomics. 2023 Jan 9;24:10. doi: 10.1186/s12864-023-09110-0 (PMC9830730; doi:10.1186/s12864-023-09110-0)

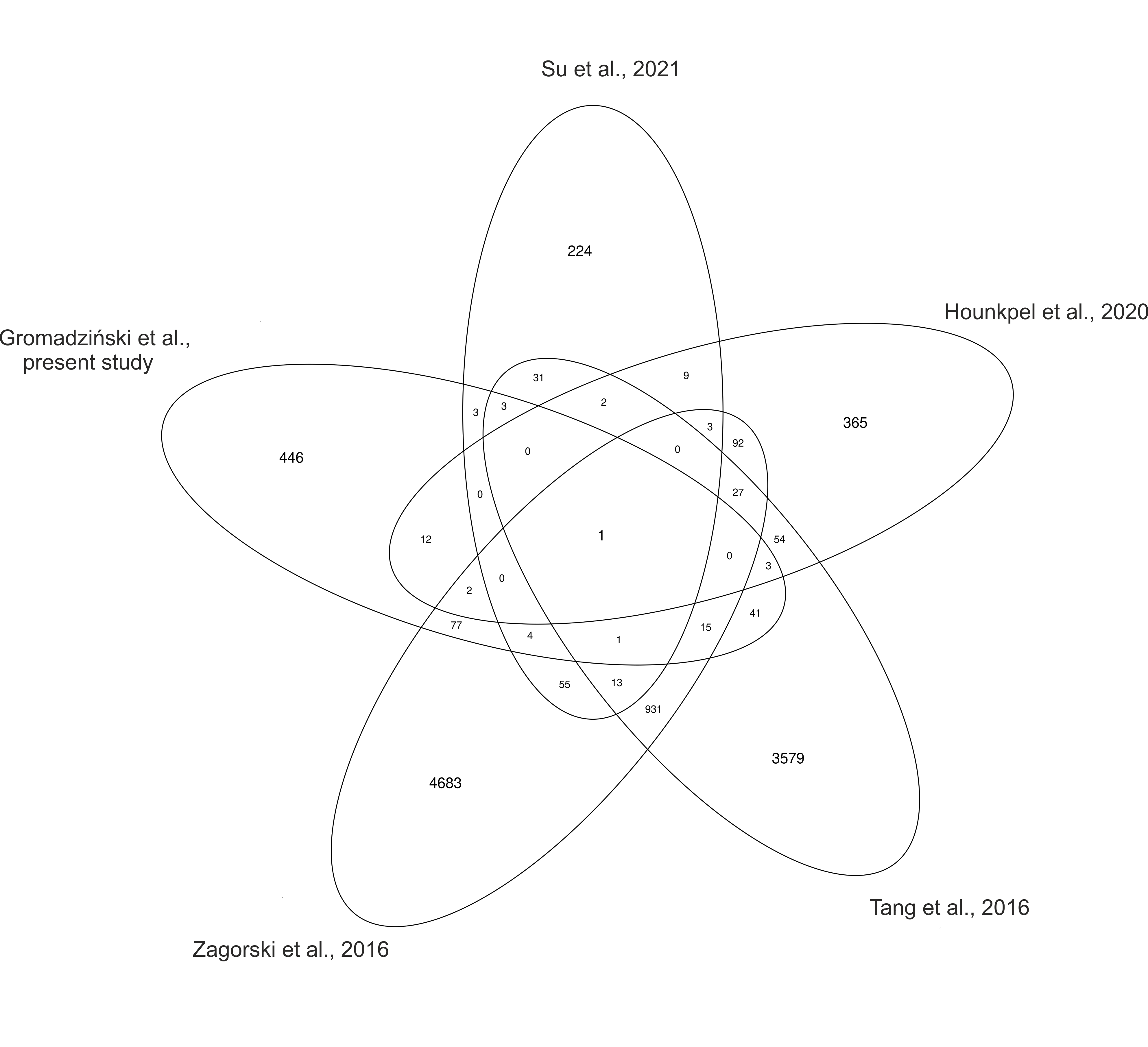

Supplement: Supplementary file 8 — Additional file 8: Figure S1. Venn diagram of DEGs comparing the obtained results with other datasets focusing on expression analysis of the DVT and PE. The numbers within the intersected ovals indicate the count of DEGs common within experiments. The outer ovals depict DEGs which are unique in the particular research. [file 12864_2023_9110_MOESM8_ESM.tif]

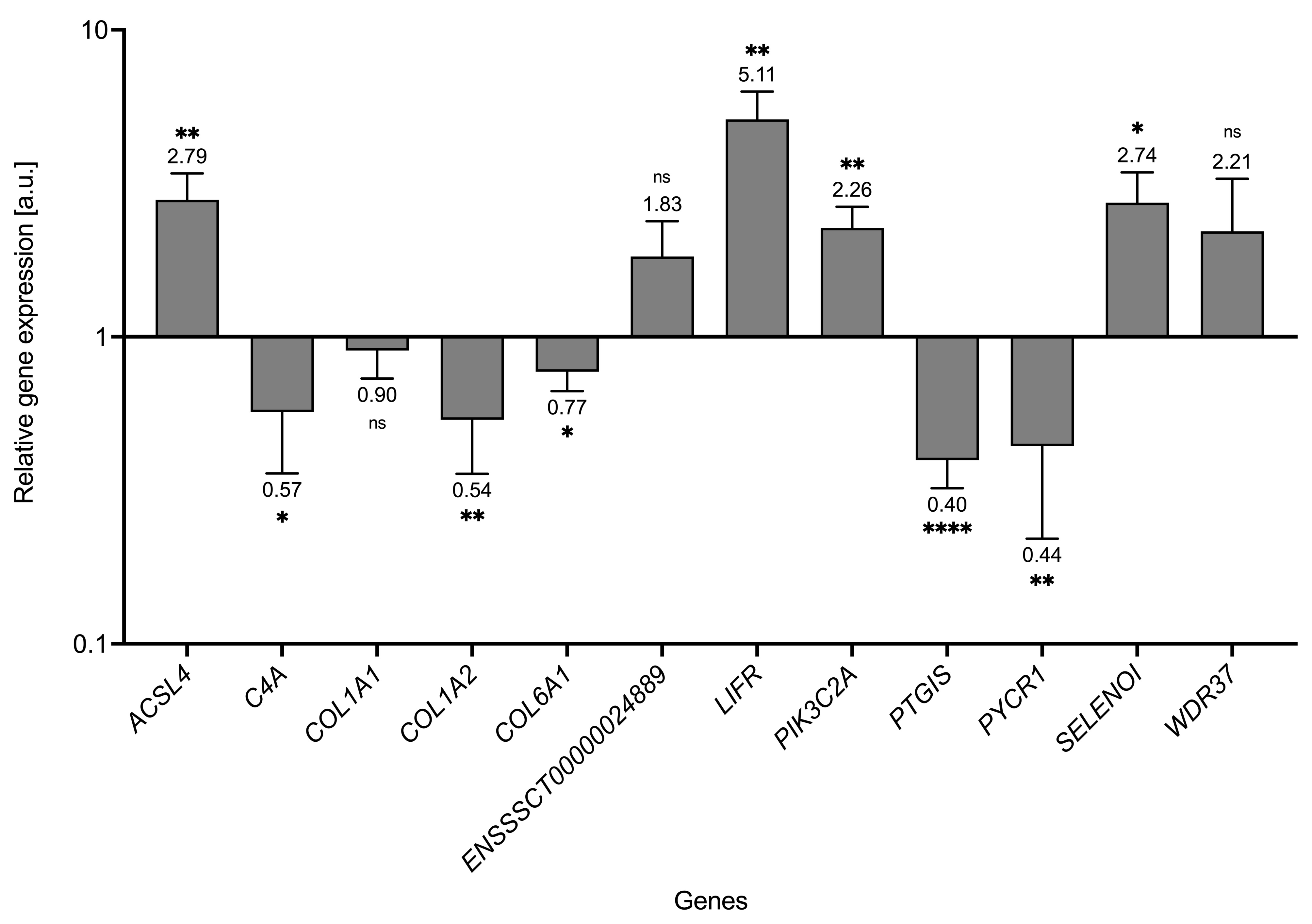

Supplement: Supplementary file 9 — Additional file 9: Figure S2. The mRNA expression of selected genes obtained using Real-Time PCR. The expression of endogenous controls is shown as normalised to a value 1, and the samples indicate the changes relative to the controls. All the relative expression units [a. u.] were presented as logarithmic values. The exact values of expression are above the bars. P-values were considered statistically significant where 0.0332 (*), 0.0021 (**) and <0.0001 (****); ns - not significant, but tended to have expression changes. [file 12864_2023_9110_MOESM9_ESM.tif]
